# Diversity of Rabies Virus Variants in Insectivorous Bats (Chiroptera: Vespertilionidae and Molossidae): An Epidemiological Study in Central Argentine Patagonia

**DOI:** 10.3390/v17060788

**Published:** 2025-05-30

**Authors:** Analía L. Giménez, Marcelo J. Zabalza, Laura P. Novaro, Gabriela A. Centurion, Melanie Y. Barrios-Benito, Ivana Moncá, Fabricio Chaar Letourneau, Román Casanovas, Susana E. Russo

**Affiliations:** 1 Centro de Investigación Esquel de Montaña y Estepa Patagónica (CIEMEP, CONICET-UNPSJB), Esquel CP 9200, Chubut, Argentina; 2 Laboratorio de Investigaciones en Evolución y Biodiversidad (LIEB, FCNyCS, UNPSJB), Esquel CP 9200, Chubut, Argentina; 3 Dirección de Laboratorio Animal (DLA), DGLyCT, SENASA, Talcahuano 1660, Martínez, Buenos Aires CP 1640, Argentina; mzabalza@senasa.gob.ar (M.J.Z.); lnovaro@senasa.gob.ar (L.P.N.); gcenturion@senasa.gob.ar (G.A.C.); mebarrios@senasa.gob.ar (M.Y.B.-B.); srusso@senasa.gob.ar (S.E.R.); 4 Laboratorio Regional Esquel, SENASA, Esquel CP 9200, Chubut, Argentina; imonca@senasa.gob.ar (I.M.); fchaar@senasa.gob.ar (F.C.L.); 5 Departamento de Zooantroponosis, División Cushamen, Secretaría de Salud del Chubut, Lago Puelo CP 9211, Chubut, Argentina; chubutzooantroponosis@gmail.com

**Keywords:** rabies, insectivorous bats, Patagonia, antigenic variants, epidemiological surveillance

## Abstract

Rabies virus (RABV) causes a fatal infection in the central nervous system of mammals. RABV circulates through two different epidemiological cycles—terrestrial and aerial—with bats being the natural reservoir of the aerial cycle. In Patagonia, only variants (V) associated with insectivorous bats have been detected. The aim of this study was to assess the diversity of circulating RABV variants in bats from Central Patagonia, Argentina. Fifty-six samples of seven bat species from eleven localities in Chubut province were analyzed using a direct immunofluorescence and biological assay, while antigenic variants were determined using an indirect immunofluorescence test. Twelve samples tested positive for RABV (>21%). Variants V4 and V6 were identified in samples of *T. brasiliensis* and *L. varius*, respectively. The remaining positive samples did not exhibit any antigenic pattern previously identified in Argentina. These samples were associated with *H. macrotus*, *H. magellanicus*, *H. montanus*, and *L. varius*. Our results confirm RABV circulation in over 71% of the bat species analyzed and in over 63% of the localities assessed. We recommend maintaining active surveillance at both local and regional levels to ensure the early detection of cases and transmission risks, which is crucial for disease prevention and control.

## 1. Introduction

Rabies virus (RABV; genus *Lyssavirus*, family Rhabdoviridae) is an etiological agent that causes a fatal infection in the central nervous system in mammals [1,2]. Among lyssaviruses, RABV was the first to be identified and remains the most widespread, capable of infecting all mammals, both wild and domestic, including humans [1,2,3,4]. RABV is primarily transmitted through the bite of an infected animal [1,5] and circulates through two distinct epidemiological cycles, classified as terrestrial (e.g., dogs, foxes, raccoons, or skunks; order Carnivora) or aerial (bats; order Chiroptera) according to their natural reservoirs [1,3]. These cycles may also be geographically described as urban or rural/wild, each encompassing both terrestrial and aerial components [3].

In Argentina, as well as throughout the Americas, only Phylogroup I has been identified in both the terrestrial and aerial cycles [3]. Likewise, two terrestrial variants (V) were found: V1, which is mainly associated with domestic dogs, and V2, associated with wild carnivores (e.g., foxes and coati) [3]. For the aerial cycle, the circulation of two variant types has been established, the first related to the hematophagous bat *Desmodus rotundus* (V3) and the second associated with insectivorous bats [1,2,3]. Among the variants related to insectivorous bats, five distinct lineages have been identified, associated with the *Tadarida brasiliensis* (Brazilian free-tailed bat; V4), *Myotis* (mouse-eared bats), *Eptesicus* (house bats), *Histiotus* (big-eared bats), and *Lasiurus* (hairy-tailed bats; V6) genera [1,2,3,4]. However, the transmission of variants from insectivorous bats to occasional hosts (spillover) has also been reported, including cases in terrestrial mammals and humans [3,4]. This factor was exemplified by two recent fatal cases in Argentina: the first occurred in a fox in 2018 (Rio Negro province), which tested positive for RABV V4, typically associated with *T. brasiliensis* [6]. The second report was a human case, also associated with V4, which was transmitted by a feral cat in 2021 (secondary transmission; Buenos Aires province) [7].

Monoclonal antibodies (mAbs) have played a key role in variant typing, significantly contributing to RABV epidemiological studies in Argentina [1,3,5,8,9]. The high specificity of these antibodies could determine the geographic origin and animal species that serve as the reservoirs of the analyzed virus [3,5,8]. Although alternative methods exist (e.g., genetic characterization and phylogenetic studies), antigenic typing using mAbs remains a valuable detection technique due to its specificity [3,10], which is why this method is still widely used in epidemiological studies [3,9].

In Patagonia, Argentina, only variants of RABV associated with insectivorous bats have been detected. Nine species of insectivorous bats are present in this region: *Histiotus macrotus* (big-eared brown bat), *H. magellanicus* (southern big-eared brown bat), *H. montanus* (small big-eared brown bat), *Lasiurus varius* (cinnamon red bat), *L. villosissimus* (hairy hoary bat), *Myotis chiloensis* (Chilean myotis), *M. levis* (yellowish myotis; family Vespertilionidae), *Eumops patagonicus* (Patagonian dwarf bonneted bat), and *Tadarida brasiliensis* (family Molossidae) [11,12,13,14]. Three of these species have distributions restricted to Patagonian environments in Argentina: *H. magellanicus*, *L. varius*, and *M. chiloensis* [11,12,13,14]. Several studies have demonstrated the circulation of different genetic variants of RABV associated with *Histiotus*, *Myotis*, and *Tadarida brasiliensis* in Patagonian provinces [1,2,3,4,5,6,15]. However, samples from Patagonian species are generally underrepresented in most studies, particularly in relation to endemic species of this region. In Chubut province, only two variants have been recorded, V4 and V6 (in the east of the province), as well as positive samples associated with *H. montanus* (in the southwest of the province) [1,3,5]. Despite the importance of understanding the mechanisms of virus maintenance, long-term field studies on viral dynamics in bat populations remain scarce overall [16] and are practically non-existent in Argentina. It is also essential to highlight the importance of correctly identifying host species in surveillance studies, as such species play a fundamental role in maintaining viral circulation and provide crucial information on disease ecology [17]. However, it is also important to consider that not all species that can contract the disease act effectively as virus reservoirs, as incidental cases or spillovers may occur [18]. Such errors can lead to misinterpretations regarding the complexity of disease epidemiology, the understanding of which is essential for the early detection of emerging rabies cycles, often associated with new or unexpected host species [17].

Due to the high lethality of RABV, epidemiological surveillance is essential for providing early warnings of case occurrences and transmission risks [3]. Likewise, recording the trends over time in different geographic areas and monitoring the variants of circulating viruses are also essential preventative and control measures, and thus contribute to public policies [3,19,20]. Under this premise, the aim of this study was to assess the diversity of circulating RABV variants in bats in northwest Chubut, as part of the National System for Passive Epidemiological Surveillance of Rabies in Argentina. Due to the lack of long-term, small-scale, systematic studies that address the local diversity of bats in Argentina, particularly in the Patagonian provinces, we believe that the information contained herein may be valuable to the relevant authorities as a tool for disease prevention and control.

## 2. Materials and Methods

### 2.1. Study Area

This study was conducted in the northwest of Chubut Province, Argentina, including samples from 11 localities within the Cushamen (Las Golondrinas [Lago Puelo]; Paraje Currumahuida [El Hoyo]; El Hoyo; Lago Puelo; Cushamen; Epuyén; Cholila) and Futaleufú (Esquel; Trevelin; Aldea Escolar; Corcovado) Departments. Localities were classified as rural (<2000 inhabitants) or urban (>2000 inhabitants) based on population size [21].

### 2.2. Samples

A total of 56 samples from seven insectivorous bat species were analyzed, including *Histiotus macrotus* (*n* = 18), *H. magellanicus* (*n* = 9), *H. montanus* (*n* = 1), *Lasiurus varius* (*n* = 3), *L. villosissimus* (*n* = 1), *Myotis chiloensis* (*n* = 8), and *Tadarida brasiliensis* (*n* = 15; see Supplemental Table S1). One specimen was submitted without taxonomic identification due to the urgency of diagnosis (Supplemental Table S1). Specimens were submitted to the Esquel Regional Laboratory (SENASA), the Department of Zooanthroponosis of Esquel (Chubut Secretariat of Health), and other regional zoonosis units.

The specimens were incorporated into Argentina’s National System for Passive Epidemiological Surveillance of Rabies during the period of 2022–2024. The specimens were considered suspected rabies cases as they were found dead in public or private settings, or had been captured by domestic animals (cats and dogs). The protocol was proposed according to the guidelines for the prevention, surveillance, and control of rabies in Argentina (Argentine Ministry of Health) [3].

The specimens were identified at the species level based on taxonomic characteristics [11,12,22]. We also determined their sex and age ranges (juvenile or adult). A brain sample was obtained from each specimen for analysis at the Department of Rabies and Small Animal Diseases (SENASA, National Rabies Laboratories Network of the Ministry of Health of Argentina) for rabies diagnosis.

### 2.3. Diagnosis and Antigenic Analysis

The suspected rabies samples were analyzed using several methods. Direct immunofluorescence (DIF) was performed using a commercial anti-nucleocapsid rabies conjugate (BIO-RAD, Marnes-la-Coquette, France). Viral isolation was conducted in vivo by preparing a 20% (*w*/*v*) suspension in phosphate-buffered saline, which was then used to intracerebrally inoculate 10 albino mice (11–14 g) and 16 suckling mice [9] per sample.

Antigenic variants were determined via indirect immunofluorescence (IIF) using a reduced panel of eight monoclonal antibodies (mAbs) [3,9,23], which was provided by the Centers for Disease Control and Prevention (CDC), Atlanta, Georgia, USA. In this way, the inoculated animals were observed for 28 days. Those that showed neurological signs compatible with rabies were euthanized (cervical dislocation) [24], and their brains were extracted for IIF [9]. Positive reactivity results were analyzed using previously described antigenic variant patterns [1,8]. Animal experiments were approved by the Institutional Committee for the Care and Use of Experimental Animals (CICUAE)—DGLyCT-SENASA. 

## 3. Results

Among the analyzed bat species, *Histiotus macrotus* and *Tadarida brasiliensis* accounted for the highest number of high-risk encounters with humans. For *H. macrotus*, the majority of cases (83.3%) were recorded in urban areas, while for *T. brasiliensis*, 60% were recorded in urban localities and 40% in rural areas. The remaining species each had fewer than 10 records. Further details on all samples analyzed (sex, age, origin, and localities) are provided in Supplemental Table S1.

**Figure 1:**
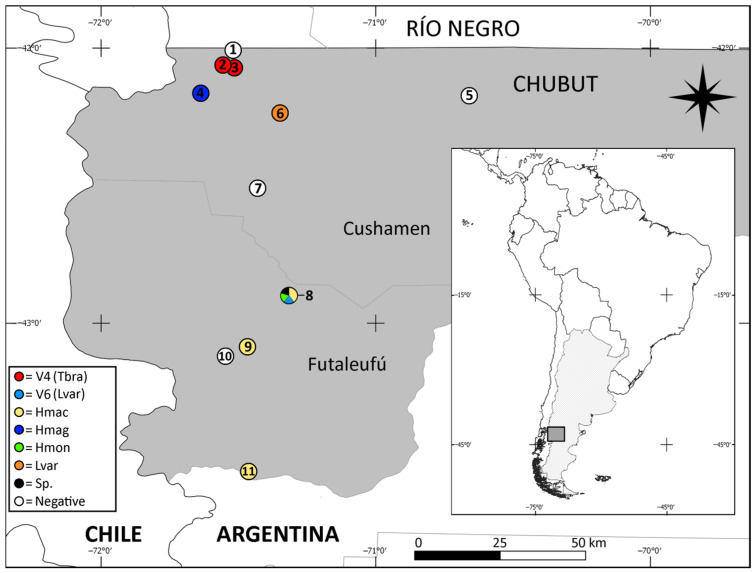
Distribution of analyzed samples in the northwest of Central Argentine Patagonia (Chubut province), including positive (colored dots) and negative (white dots) records and their associated variant (V4 and V6). 1. Las Golondrinas; 2. Paraje Currumahuida; 3. El Hoyo; 4. Lago Puelo; 5. Cushamen; 6. Epuyén; 7. Cholila; 8. Esquel; 9. Trevelin; 10. Aldea Escolar; 11. Corcovado.

A total of 12 samples tested positive for RABV, representing over 21% of the 56 samples analyzed. The positive samples corresponded to the following bat species: *H. macrotus* (*n* = 4), *H. magellanicus* (*n* = 1), *H. montanus* (*n* = 1), *L. varius* (*n* = 2), and *T. brasiliensis* (*n* = 3). The specimen without taxonomic identification also tested positive for RABV. These results indicate that over 71% of the studied species had at least one positive sample. The proportion of positive samples for each species was as follows: 66.7% for *L. varius*, 22.2% for *H. macrotus*, 20% for *T. brasiliensis*, 11.1% for *H. magellanicus*, and 100% for *H. montanus* (note that only one sample was analyzed for *H. montanus*). Virus circulation was detected in 63.6% of the studied localities (7 out of 11; see Table 1 and Figure 1). Table 1 summarizes the proportion of positive samples according to sex, age, and origin.

Antigenic variants that were identified included V4 in three *T. brasiliensis* samples and V6 in one *L. varius* sample; the remaining eight positive samples did not exhibit any previously identified antigenic patterns in Argentina. These samples were associated with *H. macrotus*, *H. magellanicus*, *H. montanus*, and *L. varius* (Table 1). Figure 1 shows the locations at which each of the variants and positive samples were recorded.

## 4. Discussion

This is the first study to analyze the diversity of circulating RABV variants in Patagonian bats on a small scale, including 87% of the species recorded in the area [8,10]. Bats are considered an important reservoir of many viruses [18,19,23,25,26]. However, the most striking epidemiological relationship with this animal is associated with its fundamental role in the spread, maintenance, and transmission of RABV [18,19,20,25]. In Argentina, insectivorous bats were long considered unimportant vectors of RABV given the country’s wide diversity of species [1,11]. As result, rabies associated with insectivorous bats, and its potential implications for public and animal health, has received minimal attention in some regions of Argentina [1], such as Patagonia. In this context, our study found a greater diversity of RABV variants in circulation than previously recorded in the analyzed area. Likewise, the prevalence percentage in the total number of samples analyzed was higher (over 21%) than that reported in previous studies. The monitoring of RABV in insectivorous bats in Argentina reported a prevalence of 3 to 5.4% [1,27], with *T. brasiliensis* accounting for 90% of the positive samples [27]. In this study, our results show that *H. macrotus* (22.2%) and *T. brasiliensis* (20%) had the highest number of positive samples. These differences between our results and previous studies may stem from the underrepresentation of Patagonian species in previous analyses that covered broader distributions in Argentina, which could lead to underestimations of the actual variability of the virus at smaller scales. Likewise, in this study, the virus was recorded in 70% of species; only *L. villosissimus* and *M. chiloensis* did not yield positive samples. Previous studies have detected RABV in *L. villosissimus*, but not *M. chiloensis*, in other regions from Argentina [1,4]. Although our results cannot confirm whether the analyzed species act as reservoirs for the virus, we confirmed the circulation of RABV among these species in the study area. Nevertheless, the role of species such as *T. brasiliensis* and *L. villosissimus* in the maintenance and spread of the virus (V4 and V6, respectively) is known in the country [3,5]. The relationship of these species with the variants recorded in the region remains to be confirmed through complementary molecular studies. In this sense, future analyses should be expanded at both the local and regional levels, including a larger number of samples, to corroborate this trend.

Regarding variant diversity, our results show that, within the aerial cycle, two well-recognized RABV variants (V4 and V6), along with one or more unidentified variants associated with *Histiotus* and *Lasiurus* species, circulated within a relatively small area (~170 km north–south), with positive cases recorded in over 60% of the assessed localities. Previous studies in Argentina identified independent lineages of RABV variants associated with different bat species (e.g., *Histiotus*, *Myotis*, *Eptesicus,* and *Lasiurus*) [1,2,4]. Therefore, our unidentified samples may be related to one of these species. In this way, our results highlight the need to more deeply explore this area of study, including molecular sequencing methods and complementary phylogenetic studies. This measure would allow us to clarify the identity of the positive samples (not associated with V6 and V4) and their relationship with the variants already detected in the area. Two of the identified variants, 4 and 6, which coincided with previous records for the Chubut province, were associated with *T. brasiliensis* [4,5], and *Lasiurus,* respectively [3]. These records were located in the east of Chubut. Therefore, our results indicate an expansion in the distribution of both variants towards the west of the province. However, when considering the migratory habits of *T. brasiliensis* [27] and *Lasiurus* species [28,29], the variants associated with these species are postulated to have a wider distribution than is currently known. Migratory bats have greater potential to spread pathogenic viruses over wider areas than locally resident species [28]. In addition, our positive results for *L. varius* represent the first report for this species. It is also important to note that one of the samples positive for *L. varius* was unrelated to V6 or to previously described virus reservoirs.

The positive samples associated with *Histiotus* species are not related to previously described variants. For *H. magellanicus*, our record is the first for the species, while for *H. macrotus*, our record represents the first for Argentina. For *H. montanus*, the results presented here may coincide with a previous record in the southwest of the province of Chubut [1]. However, this hypothesis must be confirmed by molecular sequencing. Unlike *Lasiurus* and *T. brasiliensis*, *Histiotus* species are year-round residents of the Patagonia region, and likely utilize hibernation to survive during the colder seasons [30]. This ability may also be a key factor in the maintenance and circulation of RABV. Therefore, it is extremely important to determine whether these samples belong to other known variants or those specific to this species. Bats that use hibernation present a low mortality rate during cold seasons. This feature, combined with disease characteristics such as a long incubation period, could contribute to greater survival among infected individuals from one year to the next, facilitating the infection of a new generation [19].

The diversity of variants observed in a high proportion of the species analyzed in our study could be related to the aforementioned factors as well as to the specific characteristics of bats and each species (e.g., migrants vs. residents). The colonial habits of most of these species, and the possibility of their forming mixed colonies (interspecies, including those that are migrants), could contribute to the transmission of the virus not only between individuals but also between species [19,28,31]. Movements between colonies can also expose both resident and migrant populations to new RABV variants or facilitate the exchange of variants among colonies [19,28]. Therefore, understanding the biology and ecology of bat species is essential to understand the mechanisms underlying the propagation and circulation of RABV, the dynamics of viral variants, and the likelihood of their emergence. In this regard, it is important to further investigate the variants in our study that were not associated with previously described variants to genetically characterize them and determine their relationship with those already described and their reservoirs. In this sense, we expect isolated sequences of each variant to be obtained and included in phylogenetic studies.

Our results also demonstrate the need to more deeply analyze the diversity of circulating variants at local and regional scales. Understanding the diversity of RABV variants among host species will allow us to better understand the disease, as different reservoirs have specific transmission dynamics that pose diverse risks to public health [19,20]. Predicting the spatial and temporal dynamics of interspecies transmission requires understanding how the virus is maintained in bat populations over time [16]. In addition, given previous records and the possibility of spillover [6], further studies in this area are extremely important in a region such as Patagonia, where the boundaries between urban, rural, and wild are often blurred. Therefore, maintaining the long-term systematic monitoring of insectivorous bats could be a fundamental tool for disease prevention and control, as well as to predict transmission to humans and domestic or wild animals [16], ultimately contributing to the conservation of bat populations.

## 5. Conclusions

Our study confirmed the circulation of RABV through its aerial cycle in over 70% of the insectivorous bat species analyzed (*H. macrotus*, *H. magellanicus*, *H. montanus*, *L. varius*, and *T. brasiliensis*) and in over 60% of the assessed localities in the northwestern region of Chubut province, Argentina. Our findings underscore the importance of continued surveillance in central Argentine Patagonia, given the significant prevalence of RABV among insectivorous bats. Based on these results, we recommend further studies at both the local and regional levels alongside active surveillance to ensure the early detection of cases and transmission risks, which is crucial for the prevention and control of this disease.

## Figures and Tables

**Table 1:** Positive samples tested for Patagonian bats species from northwest Chubut Province. * = unrelated to previously described virus reservoirs.

Sample	Species	Sex	Age Range	Locality	Origin	Antigenic Variant
006/22	*H.* * macrotus*	♂	Adult	Trevelin	Urban	*
008/23	*H.* * macrotus*	♂	Juvenile	Corcovado	Rural	*
012/23	*H.* * macrotus*	♀	Adult	Esquel	Urban	*
021/24	*T.* * brasiliensis*	♀	Adult	Paraje Currumahuida	Rural	V4
024/24	*L.* * varius*	♀	Adult	Esquel	Urban	V6
025/24	*T.* * brasiliensis*	♂	Adult	Paraje Currumahuida	Rural	V4
026/24	*H. montanus*	♀	Adult	Esquel	Urban	*
029/24	*H.* * magellanicus*	♂	Juvenile	Lago Puelo	Rural	*
030/24	*L.* * varius*	♂	Adult	Epuyén	Rural	*
034/24	Unidentified	-	-	Esquel	Urban	*
035/24	*H.* * macrotus*	♀	Juvenile	Esquel	Urban	*
060/24	*T.* * brasiliensis*	♂	Adult	El Hoyo	Urban	V4

## Data Availability

Data are available in the article and Supplementary Material.

## Supplementary Materials

The following supporting information is available online at https://www.mdpi.com/article/10.3390/v17060788/s1, Table S1. Samples analyzed from northwestern Chubut (Central Argentine Patagonia).
