# Supplementary material for: Diversity of Rabies Virus Variants in Insectivorous Bats (Chiroptera: Vespertilionidae and Molossidae): An Epidemiological Study in Central Argentine Patagonia
Source: Viruses. 2025 May 30;17(6):788. doi: 10.3390/v17060788 (PMC12197604; doi:10.3390/v17060788)
Supplement: Supplementary file 1 [file viruses-17-00788-s001.zip › viruses-3590362-supplementary.pdf]

viruses

MDPI

Article

Diversity of Rabies Virus Variants in Insectivorous Bats (Chiroptera: Vespertilionidae and Molossidae): An Epidemiological Study in Central Argentine Patagonia

Analía L. Giménez[1,2], Marcelo J. Zabalza[3], Laura P. Novaro[3], Gabriela A. Centurion[3], Melanie Y. Barrios-Benito[3], Ivana Monca[4], Fabricio Chaar Letourneau[4], Román Casanovas[5] and Susana E. Russo[3]

Supplementary Materials: The following supporting information can be downloaded at: www.mdpi.com/xxx/s1

Table S1. Samples analyzed from northwest of Chubut (Central Argentine Patagonia).

Sample	Species	Sex	Age range	Locality	Origin	Diagnosis
001/22	*T. brasiliensis*	♂	Adult	Paraje Las Golondrinas	Rural	Negative
002/22	*H. macrotus*	♂	Adult	Esquel	Urban	Negative
003/22	*H. macrotus*	♂	Adult	Esquel	Urban	Negative
004/22	*H. magellanicus*	♂	Adult	Epuyén	Urban	Negative
006/22	H. macrotus	♂	Adult	Trevelin	Urban	Positive
007/23	*L. varius*	♂	Adult	Esquel	Urban	Negative
008/23	H. macrotus	♂	Juvenile	Corcovado	Rural	Positive
009/23	*T. brasiliensis*	♀	Adult	Trevelin	Urban	Negative
010/23	*H. magellanicus*	♂	Adult	Trevelin	Urban	Negative
011/23	*T. brasiliensis*	♀	Adult	Trevelin	Urban	Negative
012/23	H. macrotus	♀	Adult	Esquel	Urban	Positive
013/23	*T. brasiliensis*	♀	Adult	Trevelin	Urban	Negative
014/23	*M. chiloensis*	♂	Juvenile	Trevelin	Rural	Negative
015/23	*L. villosissimus*	♂	Adult	Aldea Escolar	Rural	Negative
016/23	*H. macrotus*	♀	Adult	Trevelin	Rural	Negative
017/23	*T. brasiliensis*	♂	Adult	El Hoyo	Rural	Negative
018/23	*H. macrotus*	♀	Adult	Esquel	Urban	Negative
019/23	*T. brasiliensis*	♂	Adult	Trevelin	Urban	Negative
020/23	*H. macrotus*	♀	Adult	Esquel	Urban	Negative
021/24	*T. brasiliensis*	♀	Adult	Paraje Currumahuida	Rural	Positive
022/24	*T. brasiliensis*	♂	Adult	El Hoyo	Rural	Negative
023/24	*H. macrotus*	♂	Adult	Trevelin	Urban	Negative
024/24	L. varius	♀	Adult	Esquel	Urban	Positive
025/24	T. brasiliensis	♂	Adult	Paraje Currumahuida	Rural	Positive
026/24	H. montanus	♀	Adult	Esquel	Urban	Positive
027/24	*T. brasiliensis*	♂	Adult	El Hoyo	Urban	Negative
028/24	*H. magellanicus*	♀	Juvenile	Trevelin	Urban	Negative
029/24	*H. magellanicus*	♂	Juvenile	Lago Puelo	Rural	Positive
030/24	*L. varius*	♂	Adult	Epuyén	Rural	Positive

031/24	*H. macrotus*	♂	Adult	Trevelin	Urban	Negative
032/24	*H. magellanicus*	♀	Juvenile	Trevelin	Urban	Negative
033/24	*H. magellanicus*	♀	Adult	Esquel	Urban	Negative
034/24	-	-	-	Esquel	Urban	Positive
035/24	*H. macrotus*	♀	Juvenile	Esquel	Urban	Positive
036/24	*T. brasiliensis*	♂	Juvenile	Paraje Las Golondrinas	Rural	Negative
038/24	*H. macrotus*	♂	Juvenile	Trevelin	Urban	Negative
040/24	*H. macrotus*	♀	Adult	Esquel	Urban	Negative
041/24	*H. magellanicus*	♂	Juvenile	Esquel	Urban	Negative
042/24	*H. macrotus*	♂	Juvenile	Trevelin	Urban	Negative
043/24	*M. chiloensis*	♂	Juvenile	El Hoyo	Urban	Negative
044/24	*H. macrotus*	♂	Adult	Esquel	Urban	Negative
045/24	*H. macrotus*	♂	Adult	Cushamen	Rural	Negative
046/24	*H. macrotus*	♀	Juvenile	Trevelin	Urban	Negative
047/24	*T. brasiliensis*	♂	Adult	El Hoyo	Urban	Negative
048/24	*M. chiloensis*	♀	Juvenile	Cholila	Rural	Negative
049/24	*M. chiloensis*	♀	Juvenile	Cholila	Rural	Negative
050/24	*M. chiloensis*	♀	Juvenile	Cholila	Rural	Negative
051/24	*M. chiloensis*	♀	Juvenile	Cholila	Rural	Negative
052/24	*M. chiloensis*	♀	Juvenile	Cholila	Rural	Negative
053/24	*H. macrotus*	♀	Juvenile	Esquel	Urban	Negative
055/24	*T. brasiliensis*	♀	Adult	Lago Puelo	Urban	Negative
056/24	*M. chiloensis*	♀	Juvenile	Trevelin, route to Aldea Escolar	Rural	Negative
058/24	*H. magellanicus*	♂	Adult	Esquel	Urban	Negative
059/24	*T. brasiliensis*	♂	Adult	Trevelin	Urban	Negative
060/24	T. brasiliensis	♂	Adult	El Hoyo	Urban	Positive
062/24	*H. magellanicus*	♂	Adult	Trevelin	Urban	Negative
